# How to interpret the transmissibility of novel influenza A(H7N9): an analysis of initial epidemiological data of human cases from China

**DOI:** 10.1186/1742-4682-10-30

**Published:** 2013-05-04

**Authors:** Hiroshi Nishiura, Kenji Mizumoto, Keisuke Ejima

**Affiliations:** 1 School of Public Health, The University of Hong Kong, Level 6, Core F, Cyberport 3, Pokfulam, Hong Kong; 2 Department of Global Health Policy, Graduate School of Medicine, The University of Tokyo, Hongo 7-3-1, Bunkyo-ku, Tokyo 110-0033, Japan; 3 Institute of Tropical Medicine and the Global Center of Excellence Program, Nagasaki University, Nagasaki, Japan; 4 Department of Mathematical Informatics, Graduate School of Information Science and Technology, The University of Tokyo, 7-3-1 Hongo, Bunkyo-ku, Tokyo, 113-8656, Japan

## Abstract

**Background:**

As the human infections with novel influenza A(H7N9) virus have been reported from several different provinces in China, the pandemic potential of the virus has been questioned. The presence of human-to-human transmission has not been demonstrated, but the absence of demonstration does not guarantee that there is no such transmission.

**Methods:**

A mathematical model of cluster size distribution is devised without imposing an assumption of subcriticality of the reproduction number and accounting for right censoring of new clusters. The proportion of cases with a history of bird contact is analytically derived, permitting us to fit the model to the observed data of confirmed cases. Using contact history with bird among confirmed cases (n = 129), we estimate the reproduction number of the novel influenza A(H7N9) from human to human.

**Results:**

Analysing twenty confirmed cases with known exposure, the reproduction number for human-to-human transmission was estimated at 0.28 (95% CI: 0.11, 0.45). Sensitivity analysis indicated that the reproduction number is substantially below unity.

**Conclusions:**

It is unlikely to observe an immediate pandemic of novel influenza A(H7N9) virus with human to human transmission. Continued monitoring of cases and animals would be the key to elucidate additional epidemiological characteristics of the virus.

## Background

As the human infections with novel influenza A(H7N9) virus have been reported from several different provinces in eastern China [1], scientists, public health experts and all others concerned including the general public have started to question the pandemic potential of the virus [2]. The question largely owes to the fact that a high transmission potential could indicate a high risk of forthcoming pandemic. While the direct link between animal and human has yet to be established [3,4], the preliminary genetic analysis of the virus, the virus isolation from poultry, pigeon and quail, and the presence of a contact history with bird among a part of confirmed cases imply that a part of cases were caused by bird-to-human transmission [3-7]. Otherwise, the sources of infection have yet to be fully clarified and confirmed cases from one province have not been linked to those from other provinces [4].

A large number of traced contacts and health care workers associated with confirmed cases have not been diagnosed as infected with the virus. Thus, the presence of human-to-human transmission has not been demonstrated, but the absence of demonstration does not guarantee that there is no such transmission. In fact, there have been two family clusters reported [4]. Given that a majority of confirmed cases have been severe with unknown sources of infection and with reports from several different provinces [1,4], and considering that the confirmed cases may represent only a part of infections, it is natural to doubt if many infections are undiagnosed and if an unrecognised epidemic is going on involving frequent transmissions from human to human in the affected geographic locations.

Full clarification of the transmission potential requires additional case data and information including the results from epidemiological investigation of traced contacts [8]. However, we believe that publicly available data of the limited number of confirmed cases, coupled with the latest information of the history of contact with bird among the cases [8], have already informed us with a key insight into the transmissibility. This study aims to demonstrate that the currently available confirmed case data with a history of contact with bird can indicate that the transmission potential of the novel influenza A(H7N9) may be limited.

## Methods

### Case data

**Figure 1:**
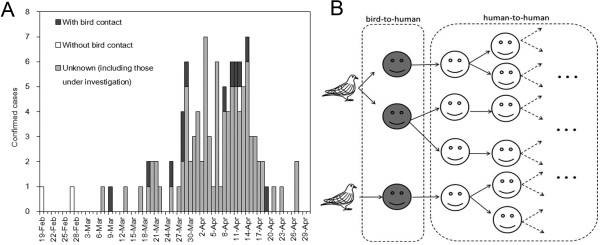
**Temporal distribution and transmission tree of the novel influenza A(H7N9). A**. Temporal distribution of confirmed cases according to the date of illness onset by history of contact with birds (n = 109 with known date of illness onset as of 1 May 2013; the dates of illness onset for other 20 confirmed cases have not been known including 1 asymptomatic infected with a history of contact with bird). Cases with a known history of contact with bird are highlighted in dark grey, while cases without contact are in white. **B**. The transmission tree that classifies cases into those caused by bird-to-human transmission (grey) and human-to-human transmission (white). A virus with substantial human-to-human transmission potential could yield many subsequent generations of cases caused by human-to-human transmission.

We analysed the confirmed case data reported by the Chinese government [9] and World Health Organization (WHO) [1]. As of 1 May 2013, a total of 129 confirmed cases including twenty four deaths have been reported from different administrative regions (provinces and cities) and Taiwan. Although frequent reports of confirmed cases took place from 31 March to the first half of April 2013, the temporal distribution of cases by date of illness onset may give a slightly different impression (Figure 1A). That is, more than 9 weeks have passed since the illness onset of the index case on 19 February 2013. Moreover, the frequency of illness onset has been diffuse in early March, although it has become more frequent to observe cases on and after 19 March 2013 than before. Other than the date of illness onset, individual case information has been accompanied by age, gender, geographic location (province or city), current clinical status (or prognosis if already known), the date of diagnosis and the presence of contact with animals including birds. Of the 129 cases, 17 have been publicly known to have had an exposure to bird, three have been known not exposed, and otherwise, the bird contact among other confirmed cases have publicly remained unknown including those under investigation. In the latest preliminary epidemiological investigation of 82 cases including 77 cases with known contact, 59 (76.6%) were revealed to have been exposed to poultry (n = 45), duck (n = 12), pigeon (n = 8) and swine (n = 4) [8]. We utilise the contact information to estimate the transmissibility.

### Mathematical model

Figure 1B shows the transmission tree that classifies cases into those caused by bird-to-human transmission and human-to-human transmission. If the human-to-human transmission potential is high yielding substantial number of human secondary, tertiary and further generations of cases, the proportion of cases without a history of bird contact among all cases should increase and approach to 100%. Here we build a mathematical model that describes this observation.

Let *k* be the number of infections caused by bird-to-human transmission. Only a part of *k* are diagnosed and reported as confirmed cases. Let *R* be the reproduction number of human-to-human transmission, representing the average number of secondary cases caused by a single primary human case during his/her entire course of infection. If *R* is above unity, the pandemic could occur with a certain probability. The possible number of generations of cases due to human-to-human transmission is denoted by *n*, and let *f*_n_ be the probability mass function of the generations that each case with a history of bird contact could have already generated. We assume that *f*_n_ follows a geometric distribution, because we assume an increasing trend in the number of primary cases with bird contact as a function of time in Figure 1A and we would like to parameterize it as a linear (exponential) growth using only one parameter while modelling it in discrete unit of time (i.e. by generation of cases). We also assume that confirmed cases with a history of bird contact developed illness proportionally to the total infected individuals caused by bird-to-human transmission (so that we can estimate the parameter *p* of the geometric distribution, *f*_n_ = (1-*p*)^n^*p* based on the time-dependent growth of confirmed cases with a history of bird contact). Due to geometric series of cases [10,11], the total number of infections caused by human-to-human transmission (*C*_hh_) is written as

(1) $$C_{\mathrm{hh}}=k\left(\sum_{n=0}^{\infty }f_{n}\frac{1-R^{n}}{1-R}-1\right),$$

while the total number of infections caused by bird-to-human transmission (*C*_hb_) is *k*. Assuming that the process of confirmatory diagnosis is independent of the source of infection (i.e. the proportion of confirmed cases with a history of bird contact among all confirmed cases is identical to that among all infected individuals), the probability of bird-to-human transmission, *q*_hb_ is given by

(2) $$q_{\mathrm{hb}}=\frac{k}{k\left(\sum_{n=0}^{\infty }f_{n}\frac{1-R^{n}}{1-R}-1\right)+k}=\frac{1}{\sum_{n=0}^{\infty }f_{n}\frac{1-R^{n}}{1-R}}.$$

If the cases with a history of bird contact are *α* times less likely to be confirmed than cases with human-to-human transmission (e.g. due to an increased likelihood to identify contacts during outbreak investigation [12]), the probability *q*_hb_ that accounts for the ascertainment bias is written as

(3) $$q_{\mathrm{hb}}=\frac{\mathrm{\alpha k}}{k\left(\sum_{n=0}^{\infty }f_{n}\frac{1-R^{n}}{1-R}-1\right)+\mathrm{\alpha k}}=\frac{\alpha }{\sum_{n=0}^{\infty }f_{n}\frac{1-R^{n}}{1-R}-1+\alpha }.$$

where *α* < 1. Suppose that there are *i* cases who had a contact with bird and *j* who had not, and that the elapsed number of generations *n*_i_ is known for each primary case *i* with a history of bird contact, the likelihood function is

(4) $$L\left(R,p;i,j,\alpha ,n_{i}\right)=q_{\mathrm{hb}}{}^{i}{\left(1-q_{\mathrm{hb}}\right)}^{j}\prod_{u=1}^{i_{0}}{\left(1-p\right)}^{n_{u}}p,$$

where *i*_0_ represents the total number of cases with known bird contact and *n*_i_ was calculated from the data as the floor (or the largest previous integer) of the difference between the date of illness onset of primary case with bird contact and the latest possible time to observe illness onset (27 April 2013) followed by the division by the mean generation time. The binomial sampling process of cases arising from a history of contact with animal reservoir was similarly modelled in a recent study of swine influenza, restricted to the case of *R* < 1 [9]. The maximum likelihood estimates of *R* and *p* were obtained by minimizing the negative logarithm of equation (4). The 95% confidence intervals (CI) were derived from the profile likelihood which exploits a likelihood-ratio test to measure the uncertainty bound.

We examined two scenarios as our baseline. As of 1 May 2013, it is known that there have been *i* = 17 confirmed cases with a history of contact with bird. Among other 112 confirmed cases, only 3 have been known not exposed. Our first scenario uses only the cases with known exposure (n = 20), namely *i* = 17 and *j* = 3 as the baseline of the first scenario. This scenario strictly adheres to the only available exposure information to assess the transmissibility from human to human. Since *i* = 17 may have included those not acquiring infection from bird (as they include severe cases), and because 3 cases without contact history might not have simply noticed the contact, we varied *i* from 10 to 19 as part of sensitivity analysis. In the second scenario, we set *i* = 99 and *j* = 30 as our baseline. This corresponds to the reported proportion of cases with bird contact [8], and the uncertainty may range from 67.1% to 86.1%. Some of *j* = 30 cases without contact information may later be revealed to have been actually exposed (e.g. without realising the exposure) [13]. Thus, we again varied *i* from 86 to 111 (which corresponds to the uncertainty bound in recent epidemiological investigation [8]).

The relative ascertainment of cases with bird contact, *α* was fixed at 1 in our baseline scenarios, while alternative scenarios should scale down *α* below 1. We examined the reproduction number with *α* < 1. To calculate *n*_i_ in the likelihood function (4), we assumed that the generation time of human-to-human transmission is a constant with the mean of 3 days [14]. As part of sensitivity analysis, we also examined *R* by varying the mean generation time from 2 to 4 days.

## Results

**Figure 2:**
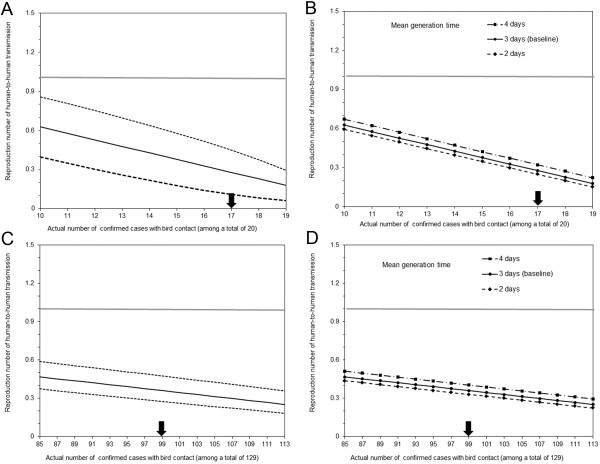
**The estimated reproduction number of human-to-human transmission for the novel influenza A(H7N9). **Each line shows the estimated reproduction number of human-to-human transmission for novel influenza A(H7N9). If the reproduction number exceeds 1, it indicates that a pandemic in human population can be caused with a certain probability. Our estimates depend on the proportion of cases caused by bird-to-human transmissions among a total of confirmed cases, and thus, the actual number of confirmed cases with bird contact. As of 1 May 2013, seventeen cases have been reported to have had contact with bird. Panels **A **and **B **show the results from the first scenario in which we analysed only the cases with a known history of exposure (i.e. 17 contacts with bird and 3 cases without bird contact). Panels **C **and **D **show the results from the second scenario, which adhered to the latest contact tracing information [8], considering that 99 out of 129 confirmed cases had a history of exposure to bird. Panels **A** and **C** additionally show the 95% confidence intervals in dashed lines, while panels **B** and **D** vary the mean generation time from 2 to 4 days (baseline: 3 days). In all panels, solid line represents the maximum likelihood estimates of the reproduction number for human-to-human transmission for the baseline scenario. The horizontal grey line corresponds to the reproduction number at unity above which a pandemic could occur. An arrow in each panel on the horizontal axis points the baseline assumption of the number of contacts with bird.

Figure 2 shows the estimates of the reproduction number for human-to-human transmission of the novel influenza A(H7N9). Based on confirmed cases with known contact with bird, the mean number of generations that may have already elapsed was estimated at 8.0 (95% CI: 4.6, 15.4) using the mean generation time of 3 days. The mean number of generations of cases already elapsed was estimated at 12.1 and 5.8 by using different mean generation time of 2 and 4 days, respectively.

Using only 20 known exposures in the first scenario, the reproduction number was estimated to be 0.28 (95% CI: 0.11, 0.45). The upper confidence intervals were well below unity for the examined scenarios. With *α* < 1, the reproduction number appears to be even smaller (Results not shown). Figure 2B examines the sensitivity of the reproduction number to the mean generation time. If the generation time is longer, the reproduction number is estimated as bigger.

Figure 2C and 2D show the estimates of the reproduction number in our second scenario. In the worst case scenario with *i* = 99 and *j* = 30, the reproduction number was estimated at 0.36 (95% CI: 0.33, 0.40). If more bird contacts are revealed in future, the reproduction number is estimated to be smaller (Figure 2C). For instance, if 6 more cases are revealed to have been exposed to poultry, the maximum likelihood estimate of *R* is 0.31.

## Discussion

We have demonstrated that the proportion of confirmed cases with a history of bird contact can inform the magnitude of the transmission potential, showing that the reproduction number for human-to-human transmission of the novel influenza A(H7N9) is well below unity. Our finding is consistent with the lack of demonstration of human-to-human transmission among traced contacts of confirmed cases. Since the absence of secondary transmission during contact tracing practice can only indicate that the human transmission may be limited (because the quantitative objectiveness of traced “contact” is limited), our finding of *R* < 1 is significant in that it can quantitatively imply that it is unlikely to observe an immediate pandemic with substantial transmissions from human to human.

Our modelling exercise emphasises that a high proportion of cases with a history of bird contact among all cases is regarded as good news (to anticipate the pandemic). If a pandemic is very likely, cases caused by human-to-human transmission should become widespread in a short period of time, and cases with bird contact may not be even visible among the total number of confirmed cases (e.g. recall the confirmed case data of influenza A(H1N1-2009) which was filled with those caused by human-to-human transmission even during the early stage). As examined earlier for a different influenza [12], we have shown that the information of the proportion with a history of bird contact can be explicitly translated to an estimate of *R*. Among published studies of the transmissibility of avian and swine influenza in humans [12,15-18], only Cauchemez et al. [12] utilised the information of the number of reservoir-borne transmission among all cases to estimate the transmissibility. While Cauchemez et al. [12] examined the similar data without censoring and specifically for a subscritical process (i.e. under an assumption of *R* < 1) with the mean cluster size of 1/(1-*R*), the present study further accounted for right censoring of the clusters of cases (and also the right censoring of the contact investigation result) without imposing an assumption of subcriticality (i.e. *R* can take the value either below or above unity depending on the data). For public health practice, Cauchemez et al. [12] and our modelling exercise suggest that the traced information of contact with animals would be critical in epidemiological assessment of the pandemic potential especially shortly after the emergence in human population. An updated news of exposure to poultry among a substantial fraction of cases [8] indicates that the exposure to poultry has been very likely underreported in real time (i.e. during the course of an outbreak). To yield additional insights into the epidemiological characteristics, the animal contact should be explored more in detail and routinely reported in a timely manner, including the time and place of contact as well as detailed species of animals.

Our finding of *R* < 1 indicates that the continued monitoring of cases in future is even more important than in the past, because the majority of cases may have been exposed to bird without realising the exposure. Identifying the exact sources of infection that have successfully generated the spatially diffuse outbreak would be the key to bring the ongoing outbreak under control. In this context, routine surveillance and monitoring of animal influenza including those conducted at poultry market are recommended to help elucidating the transmission dynamics [19].

Three limitations should be noted. First, except for ascertainment bias, we assumed that the proportion of bird contact among confirmed cases mirrors that of all infected individuals. Our analysis assumed that exposure to poultry among confirmed cases is a good proxy for actual bird-to-human infection; our estimates of *R* may be biased if this assumption is violated. Second, our model rested on simple homogeneous mixing assumption to gain some gut feeling of the transmissibility (e.g. below or above unity) based on limited confirmed case data. The proposed simple model ignored a well-known variation in the number of secondary cases at an individual level (i.e. the variance of the offspring distribution), which could have led our CI to be unrealistically narrow [20,21]. Third, as was indicated by Cauchemez et al. [12], the estimate of the reproduction number using the fraction of cases with a history of reservoir contact may be biased upward if some cases are detected through outbreak investigation. That is, our estimate of *R* derived from 20 cases with known contact may have been even an overestimate, because human-to-human transmissions are more likely to be detected than bird-to-human transmissions during contact tracing practice. This point supports our main conclusion of *R* < 1.

Despite these limitations, we believe that the present study has successfully extracted the information of transmissibility. It is unlikely that the pandemic will occur immediately, but continued monitoring of cases and animals would be the key to elucidate additional epidemiological characteristics of the novel influenza A(H7N9) virus.

## Conclusions

We proposed a mathematical model of cluster size distribution of influenza without imposing an assumption of subcriticality accounting for right censoring of new clusters of cases. Using contact history with bird among confirmed cases (n = 129), we estimate the reproduction number of the novel influenza A(H7N9) from human to human. Analysing twenty confirmed cases with known exposure, the reproduction number for human-to-human transmission was estimated at 0.28 (95% CI: 0.11, 0.45). Sensitivity analysis indicated that the reproduction number is substantially below unity. It is unlikely to observe an immediate pandemic of novel influenza A(H7N9) virus with substantial human-to-human transmissions.

## Competing interests

The authors declare that they have no competing interests.

## Authors’ contributions

HN conceived the study idea, constructed the epidemiological model and implemented the statistical analysis. KM collated, summarised and updated the data and KE elaborated the model. HN, KM and KE jointly drafted the manuscript. All authors approved the final version of the manuscript.
